# Xanthogranulomatous Pyelonephritis: Synchronous Upper and Lower Gastrointestinal Bleed

**DOI:** 10.1177/2324709619842899

**Published:** 2019-05-01

**Authors:** Muhammad Usman Mirza, John Van Taunay, Muhammad Waleed, Shiva Shankar Vangimalla, Sharana Hegde, Muhamad Alhaj Moustafa

**Affiliations:** 1 MedStar Washington Hospital Center, Washington, DC, USA; 2 Aga Khan University, Karachi, Pakistan; 3 Mayo Clinic, Jacksonville, FL, USA

**Keywords:** xanthogranulomatous pyelonephritis, gastrointestinal bleed, reno-colic fistula, reno-gastric fistula, nephrectomy

## Abstract

Xanthogranulomatous pyelonephritis (XGP) is a rare chronic granulomatous destructive process of the renal parenchyma. It is caused by a chronic inflammatory process due to recurrent urinary tract infections and/or obstructing renal calculi. Rarely, it presents with advanced complications including abscesses and fistula formations. In this article, we report a unique presentation of XGP with simultaneous upper and lower gastrointestinal bleeding in the setting of XGP with reno-gastric and reno-colic fistulas.

## Introduction

Xanthogranulomatous pyelonephritis (XGP) is a rare histological form of chronic pyelonephritis with an incidence of 1.4 cases/100 000 person per year.^1^ XGP is typically a unilateral process affecting only one kidney and is more common in middle-aged women.^1^ It was first described by Schlagenhaufer in 1916.^2^ XGP is characterized by renal parenchymal tissue destruction due to infiltrative process with lipid-laden macrophages.^1,3^ The precise causal etiology of XGP remains unknown. XGP is postulated to result from misdirected chronic activation of macrophages against bacteria.

Clinical features are variable, with nonspecific symptoms of fever, flank pain, fatigue, malaise, urinary symptoms, and weight loss. The initial diagnosis of XGP is challenging because the above-mentioned symptoms and laboratory abnormalities as well as the radiologic findings could resemble those of renal cancer.

Many cases of extrarenal involvement were reported in the literature. Extensions of the disease into adjacent organs and fistulas formations connecting the urinary tract with proximate organs are reported manifestations of XGP. Because of complications like fistula formation, XGP becomes even more difficult to diagnose. Fistulas connecting the kidney to the colon, jejunum, duodenum, bronchus, diaphragm, thorax, psoas muscle, and skin were reported.^4-12^

There are no reported cases of concurrent reno-gastric and reno-colic fistulas. Herein, we describe a unique case of reno-gastric and reno-colic fistulas in a patient with history of left renal calculus presenting with simultaneous upper and lower gastrointestinal bleed.

## Case Description

**Figure 1.**
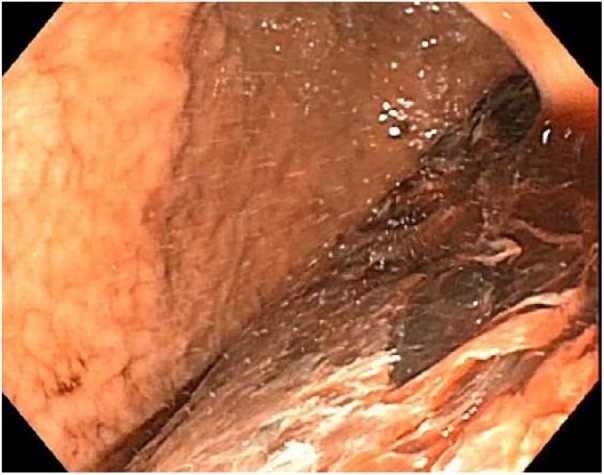
Upper endoscopy with blood clot in the stomach.

A 62-year-old female with history of left renal calculus presented to the emergency department with fatigue, syncope, 3 episodes of hematemesis, and 2 episodes of melena over the past 24 hours. Physical examination revealed an afebrile healthy female, without abdominal or flank tenderness. Her presenting hemoglobin (hgb) was 8.2 g/dL without leukocytosis. No urinalysis was obtained due to absence of any urinary symptoms. At this point, she did not have any symptoms of pyelonephritis. She was admitted to the intensive care unit, where her hematemesis continued. Repeat hgb after 1 day dropped to 6.6 g/dL. No abdominal imaging was obtained. Bedside esophagogastroduodenoscopy (EGD) revealed a large amount of clotted blood in the stomach, unamenable to lavage (Figure 1). Continued hematemesis prompted left gastric arterial embolization; however, the patient continued to have hematemesis. Repeat EGD after 4 days revealed persistent fresh blood in the stomach despite lavage. The fundus and body were empirically injected with epinephrine to achieve hemostasis. Her hgb stabilized and she was discharged home after a few days.

**Figure 2.**
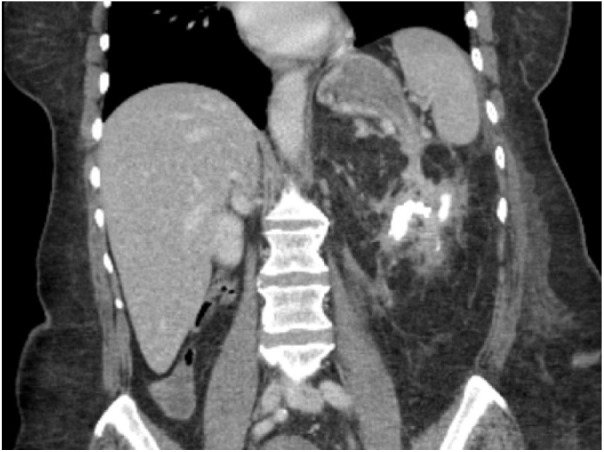
Computed tomography scan of abdomen and pelvis with intravenous contrast showing left renal calculus and reno-gastric fistula.

**Figure 3.**
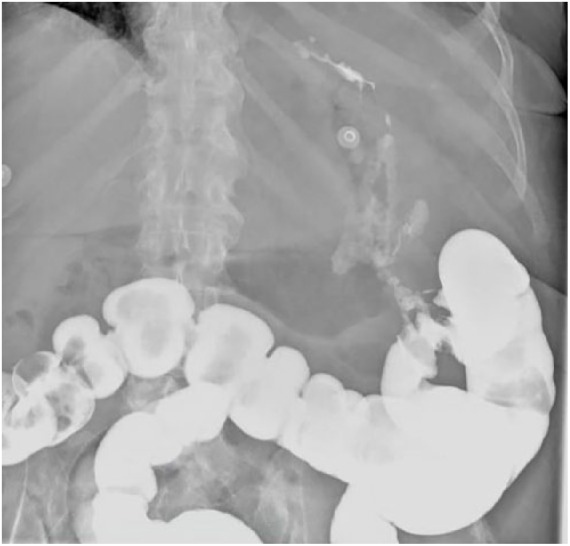
Barium enema showing contrast extravasation from the splenic flexure.

During a follow-up clinic visit after 4 weeks, she was found to have a left flank pain, fever, headache, and nausea. Patient’s hgb was 5 g/dL with positive fecal blood test. In the interim, she had intermittent melanotic stool with no hematemesis. She was readmitted to the hospital where an EGD showed a fistulous tract draining pus into the gastric fundus. A colonoscopy revealed a fistulous tract draining pus and blood into the descending colon. Epinephrine was injected and hemostasis was achieved. Gastric biopsy showed mild chronic gastritis, reactive epithelial changes, and negative for malignancy. A computed tomography (CT) scan of the abdomen and pelvis with intravenous contrast showed a left renal staghorn calculus, a peripancreatic and perirenal fluid collection suspicious for abscesses, and possible presence of fistulous tracts connecting the perirenal fluid collection to the gastric wall and the descending colon (Figure 2). A barium enema demonstrated a fistula at the level of splenic flexure with contrast extravasation into the left upper quadrant of the abdomen (Figure 3). Serum amylase, lipase, carcinoembryonic antigen, and carbohydrate antigen 19-9 were normal. Urinalysis was suggestive for urinary tract infection, and urine culture grew *Enterococcus faecalis*. The patient was started on intravenous ceftriaxone and later switched to intravenous gentamicin once the sensitivities came back.

The patient underwent exploratory laparotomy with complete left nephrectomy, complete splenectomy, distal pancreatectomy, partial colectomy of the descending colon, and repair of the reno-gastric fistula. The pathology report from the left kidney confirmed granulomatous inflammation with the presence of multinucleated giant cells consistent with the diagnosis of XGP. A follow-up CT scan of the abdomen and pelvis after 2 weeks did not show recurrence of any abscess or fistulas. After successful treatment, her hgb improved and she was discharged home with an uncomplicated postoperative course.

## Discussion

XGP is a chronic debilitating condition that is rarely fatal. It has been associated with obstruction of the urinary tract. In a report of 36 cases of XGP, 89% were caused by obstruction while 11% had no clear etiology.^13^ Anemia, leukocytosis, elevated liver enzymes, and elevated creatinine are common laboratory abnormalities seen in these patients.^3,13-15^ The most common reported infectious organisms are *Escherichia coli* and *Proteus mirabilis*.^1,13^ This disease can be suspected on radiographic findings but cannot be confirmed. CT scan remains the imaging modality of choice for XGP; irregular collecting systems and cystic areas presenting as a “bear paw” sign are characteristic of this disease.^16^ A bear paw sign refers to the cross-sectional appearance of the kidney, which occurs when the renal pelvis is contracted and the calyces are dilated, mimicking the toe-pads of the paw. Even biopsy can be inconclusive for XGP, as it can miss the classic appearance of granulomatous tissue laden with lipid-filled macrophages and necrosis surrounding calculi in kidneys.^16^

XGP could be either confined to kidneys or involve adjoining organs and organs related by sharing fascial planes. Malek and Elder, therefore, classified this disease into 3 categories according to the extent of spread of the disease: stage 1, inflammatory process limited to kidney; stage 2, inflammatory process confined to kidney and perinephric fat; and stage 3, inflammatory process spread to retroperitoneal space and surrounding structures.^14^ Treatment in most cases is removal of the diseased kidney and surrounding involved tissue.^14^

Our case was a diagnostic challenge due to its unusual presentation. Although, reno-colic fistulas were reported sporadically, there was only one reported case of reno-gastric fistula due to XGP in the literature.^17^ Fistula formation in the setting of XGP further complicates its diagnosis. Our patient presented with concomitant upper and lower gastrointestinal bleeding from reno-gastric and reno-colic fistulas, respectively. In our case, staghorn calculi possibly obstructed the kidney and led to chronic infection and inflammation with subsequent fistula formation. Although *Escherichia coli* and *Proteus mirabilis* are the common infectious organisms involved, our patient’s urine culture was positive for *E faecalis*. The causal association is unclear as to whether the patient was infected with *E faecalis* before fistulous tract formation or did *E faecalis* translocate from gastrointestinal tract to kidney after the fistula formation.

XGP is a difficult disease to diagnose especially in the setting of fistula formations. Recurrent urinary tract infections with the above-mentioned nonspecific symptoms should promote appropriate workup to rule out malignancy and XGP. Due to the nonspecific signs and symptoms of XGP, radiographic signs like barium extravasation into the renal pelvis may offer clues to diagnose fistulas related to the XGP disease. In conclusion, unexplained fistulous tracts maybe the presenting problem of an underlying XGP.
